# Needed Reform in Asylum Management, and the Lunacy Laws of Texas

**Published:** 1911-10

**Authors:** 


Needed Reform in Asylum Management, and the Lunacy Laws of Texas.—Quite a breeze has been sprung by a newspaper account of a paper read at a meeting of the Dallas County Medical Society by Mr. Wilkinson, an attorney for the Texas State Medical Association, and a newspaper controversy resulting therefrom. Mr. Wilkinson says there are people in the asylum who are not insane, and points out that persons accused of lunacy are arrested, tried and sentenced by a jury picked up on the street, and that Texas needs a lunacy commission. All these things are so patent that the wonder is that it has attracted any attention.

All readers of the Texas Medical Journal will recognize in the above exposé the contention of its editor for something like twenty-five years that there exists just the evil Mr. Wilkinson speaks of. For a quarter of a century the "Red Back" has "harped on" this needed reform, untili with its readers, it is threadbare. We have piped, but no one has danced. Maybe Mr. Wilkinson—if he shoots at the toes of ye solons, like the bad men of the West do at those of the tenderfoots—he may make 'em dance. *Nous verrons.*

